# Cloning, bioinformatics analysis and expression of the cysteine dioxygenase type 1 (CDO1) gene in domestic yak

**DOI:** 10.3389/fvets.2024.1488782

**Published:** 2024-10-18

**Authors:** Yuxin Fu, Jiuru Yan, Lan Lan, Huizhu Zhang, Peng Wang, Yaying Wang, Xianrong Xiong, Jian Li, Honghong He

**Affiliations:** ^1^Key Laboratory of Qinghai-Tibetan Plateau Animal Genetic Resource Reservation and Utilization, Ministry of Education, Chengdu, China; ^2^Key Laboratory of Animal Medicine at Southwest Minzu University of Sichuan Province, Chengdu, China; ^3^College of Animal and Veterinary Sciences, Southwest Minzu University, Chengdu, China; ^4^Animal Husbandry Science Institute of Ganzi Tibetan Autonomous Prefecture, Kangding, China

**Keywords:** yak, cloning, bioinformatics analysis, ovary, CDO1

## Abstract

**Introduction:**

The CDO1 gene is an important gene in the taurine synthesis pathway and has been observed to have high expression in ovaries of female mammals. This study aims to explore the conservation of CDO1 gene in domestic yaks, as well as to examine the fundamental characteristics of CDO1 gene and its expression in female yaks.

**Methods:**

Ovarian samples were collected from yaks in the follicular phase, luteal phase and gestation period in this experiment, and their total RNA and protein were extracted. Then Polymerase Chain Reaction (PCR) and bioinformatics online software were used to clone and analyze the CDO1 gene. The relative expression of CDO1 in yak ovaries was detected by Quantitative Real-time PCR (RT-qPCR) and Western blotting. The distribution and localization of CDO1 protein in ovary were detected by immunohistochemistry.

**Results:**

We have successfully cloned the coding region of CDO1 gene in yak. The results showed that the CDS region of CDO1 gene was 603 bp, encoding 200 amino acids, and was a relatively stable hydrophilic protein. CDO1 is relatively conservative in species evolution. The protein encoded by CDO1 gene does not have a signaling peptide or a transmembrane structure. It is a protein that is not involved in transmembrane transport and is mainly located in the cytoplasm. The secondary structure of the protein is dominated by the random coil. CDO1 is estimated to interact with 10 proteins. The results of RT-qPCR and Western blotting showed that the CDO1 gene exhibited the highest expression in the ovary during the luteal phase and the lowest expression in the ovary during the follicular phase (*P* < 0.01). The results of immunohistochemistry showed that CDO1 was mainly expressed in granular cells, theca cells and lutein cells of ovarian tissue.

**Conclusion:**

These results suggest that the CDO1 gene has undergone minimal evolutionary changes during the course of animal evolution. The results provide a reference for further investigation of the function of CDO1 gene in reproduction and production in yaks.

## 1 Introduction

The yak (*Bos grunniens*), an important animal resource living above 3,000 m on the Qinghai-Tibet Plateau, is one of the main economic resources for local herders (1). Yaks breed seasonally, with the breeding season occurring between July and October. The estrous cycle lasts 19–21 days, resulting in one litter every 2 years or two litters every 3 years. After long-term natural selection, artificial domestication and other factors, domestic yaks have shown relatively stable and unique genetic characteristics (2, 3). However, the natural reproductive efficiency of domestic yaks is low due to reasons such as the harsh plateau environment, insufficient breeding management and the retarded growth and development of yaks (4). Therefore, it seriously restricts the development of the yak industry. Improving yak fertility through modern breeding technology is an important part of the efficient development of the yak industry. Taurine is one of the most abundant free amino acids in mammals (5), and it is most abundant in the brain, ovary, uterus, heart, liver and other organs of mammals. Taurine has a wide range of biological functions *in vivo*, which can regulate cell osmotic pressure, stabilize cell membrane, remove oxygen free radicals, and resist oxidation, as well as regulate intracellular calcium channel activity to maintain calcium homeostasis and regulate nerves (6). Maintenance of taurine levels in most mammals depends on active intake of taurine from the diet and endogenous synthesis of taurine (7), which includes an important enzyme: cysteine dioxygenase.

Cysteine dioxygenase (CDO) is a key enzyme in taurine synthesis and is expressed in liver, adipose tissue, pancreas, kidney, lung and reproductive system. The liver is the major site of CDO synthesis. Under physiological conditions, CDO can catalyze L-cysteine into cysteine sulfinic acid (CSA), CSA is further broken down into taurine and sulphate (8), which reduces the neurotoxicity and cytotoxicity caused by cysteine accumulation in the body and reduces the risk of related diseases (9). There are two types of CDO, a cytoplasmic CDO (Cysteine dioxygenase type 1, CDO1) and a membrane-bound CDO (CDO2). CDO1 is a mononuclear non-hame metalloproteinase belonging to the cupin superfamily. CDO1 is mainly expressed in adipose tissue, liver, brain, small intestine, lung and kidney (8, 10). CDO1 plays an important physiological role in mammals, such as regulating the stability of REDOX state *in vivo*, participating in the metabolism of amino acids, lipids (11) and bile acids, and regulating osteoblast differentiation (12).

CDO1 also plays an active role in mammalian reproduction. CDO is involved in cysteine metabolism and taurine synthesis, and is the rate-limiting enzyme in taurine synthesis. Studies by Ueki I and others have shown that CDO^−/−^ mice have higher postpartum mortality and growth defects, and their taurine levels were deficient. Knocking out the CDO1 gene in mice resulted in impaired taurine synthesis. Supplementation of taurine to CDO^−/−^ mice with taurine did not improve pup survival (13). In addition, sperm from CDO^−/−^ mice were severely deficient *in vitro* fertilization ability and sperm morphology was distorted (14). CDO was also highly expressed in the mouse ovary and uterus (15, 16). Taurine levels in the serum and uterine tissue of CDO^−/−^ mice were severely reduced, resulting in implantation defects and a severe reduction in fertility, and supplementation of taurine partially repaired the embryo implantation defects in CDO KO mice. The study by Di Zhang et al. (15) confirmed that taurine and CDO are involved in embryonic implantation in mice by participating in E_2_-ERα and P_4_-PR signaling pathways. Rare ginsenosides (RGS) have a therapeutic effect on cyclophosphamide-induced reproductive tract damage in female rats, and the metabolomics results indicate that the therapeutic effect of RGS are related to taurine and hypotaurine metabolism, etc., thereby ameliorating cyclophosphamide-induced reproductive tract damage in female rats (17). In addition, taurine levels were higher in small and medium than large ovarian follicles in buffalo. Taurine is positively correlated with ovarian follicular testosterone levels and negatively correlated with ovarian follicular estradiol levels (18). Therefore, synthesizing CDO and endogenous taurine is essential for embryo implantation and animal reproduction maintenance. In conclusion, CDO1 plays an important role in maintaining normal physiological functions of animal reproductive organs.

However, the cloning and potential biological functions of the yak CDO1 gene have not been reported, and it is not clear whether CDO1 is present in yak reproductive organs and is involved in the reproductive process. This study aimed to clone the yak CDO1 gene, analyze its biological characteristics, and determine its expression and localization in yak ovarian tissues during different reproductive cycles, in order to provide a basis for further research on the regulation of the CDO1 gene in yak reproduction.

## 2 Materials and methods

### 2.1 Sample collection

All research animals were acquired, retained, and killed with a humane method in compliance with local laws and regulations of Sichuan province, China. Ovarian tissues from 5 healthy female yaks in estrus and pregnancy aged 4–5 years old were collected from Hongyuan County, Aba Tibetan and Qiang Autonomous Prefecture, Sichuan Province. After slaughter, the ovarian tissue was clipped with sterile scissors, and the surrounding connective tissue and fat were removed, rinsed with normal saline, and quickly placed in liquid nitrogen for preservation. After transporting to the laboratory, part of the ovarian tissues were stored in an ultra-low temperature refrigerator at −80°C, and the other part were trimmed into about 1 cm^3^ tissue blocks, which were fixed in 4% paraformaldehyde for tissue sections.

### 2.2 Tissue RNA extraction and reverse transcription

Total RNA was extracted from ovarian tissues at the follicular, luteal and gestational stages according to the instructions of the microsample total RNA extraction kit (Transgen, ET111, Beijing, China). Then the extracted RNA was reverse-transcribed into cDNA according to the instructions of the reverse transcription kit (Vazyme, R323, Nanjing, Jiangsu, China). Finally, the cDNA was stored in a refrigerator at −20°C.

### 2.3 Cloning of the yak CDO1 gene

The sequences of the CDO1 gene (NM_001034465) and *β*-actin gene (NM_173979) of *Bos taurus* were retrieved from the GenBank database, and 3 pairs of primers were designed using Primer Premier 5.0 software, the primer sequences are shown in Table 1. They were used to amplify the yak CDO1 gene sequence, *β*-actin primers were used as internal reference gene detection primers, and the synthesis of primers was completed by Sangon Biotech (Shanghai) Co., Ltd. PCR amplification was performed using ovarian tissue cDNA as a template. The PCR reaction system was 20 μL: 10 μL of 2× Rapid Taq Master Mix (Vazyme, P222-01, Nanjing, Jiangsu, China), 1 μL each of forward and reversed primers, 1 μL of cDNA, and 7 μL of ddH_2_O. The reaction procedure is as follows: pre-denaturation at 95°C or 30 min, 35 cycles were set at 95°C for 30 s, 60°C for 30 s and 72°C for 1 min; then extension at 72°C for 15 min, finally storage at 4°C. The products were detected by 1.5% agarose gel electrophoresis and showed clear and bright bands. The PCR products were sent to Sangon Biotech (Shanghai) Co., Ltd. for the cloning of the yak CDO1 gene.

**Table 1:** Primers information.

Primer	Primer sequences(5’to 3′)	Annealing temperature / °C	Product size / bp	Utilization
CDO1	F1:CAGTTCCTCCTCCATCGC	60	667	cloning
	R1:GCCCCTTAGTTGTTCTCC			
CDO1	F2:CGACTCCCACTGCTTTCTGA	58	187	RT-qPCR
	R2:GGCAGGCTCTGTATGGCTAA			
*β-actin*	F3:CTTCGAGCAGGAGATGGC	58	227	RT-qPCR
	R3:CCGTGTTGGCGTAGAGGT			

### 2.4 Bioinformatics analysis of CDO1 gene in yaks

Through the splicing of DNAMAN software, we obtained the sequence of the yak CDO1 gene. Online software was used to analyze the biological characteristics of the CDO1 gene, including open reading frame prediction, sequence alignment, protein physicochemical property analysis, protein structure and protein interaction network prediction, and the phylogenetic tree was constructed by MEGA 11.0 software. The bioinformatics analysis software and its applications are listed in Table 2.

**Table 2:** Bioinformatics analysis softwares and applications.

Softwares	Websites	Applications
ORF Finder	https://www.ncbi.nlm.nih.gov/orffinder/	Open reading frame analysis
BLAST	https://blast.ncbi.nlm.nih.gov/Blast.cgi	Sequence comparative analysis
ProtParam	https://web.expasy.org/protparam/	Analysis of physical and chemical properties of proteins
ProtScale	https://web.expasy.org/protscale/	Analysis of hydrophilic and hydrophobic properties of proteins
SignalP 4.1	https://services.healthtech.dtu.dk/services/SignalP-4.1/	Protein signal peptide analysis
TMHMM	https://services.healthtech.dtu.dk/services/TMHMM-2.0/	Protein transmembrane structure prediction
PSORT II	https://www.genscript.com/psort.html	Prediction of protein subcellular localization
NetOGlyc	https://services.healthtech.dtu.dk/services/NetOGlyc-4.0/	Protein glycosylation site
NetPhos	https://services.healthtech.dtu.dk/services/NetPhos-3.1/	Protein phosphorylation site
SOPMA	https://npsa-prabi.ibcp.fr/cgi-bin/npsa_automat.pl?page=/NPSA/npsa_sopma.html	protein secondary structure prediction
PHYRE2	http://www.sbg.bio.ic.ac.uk/phyre2/html/	Protein tertiary structure prediction
STRING 10.5	https://www.string-db.org/	Protein interaction network prediction

### 2.5 Quantitative real-time PCR (RT-qPCR)

Specific primers were designed according to the yak CDO1 gene sequence (Table 1), and RT-qPCR was used to detect the expression of CDO1 gene in ovarian tissues of female yaks during different reproductive cycles. The housekeeping gene *β*-actin was used as an internal reference gene in all assays, and a negative control assay containing all of the above reaction mixtures but no cDNA templates was included in each RT-qPCR assay. The reaction system was 20 μL: 10 μL of 2× ChamQ SYBR qPCR Master Mix (Vazyme, Q311-02, Nanjing, Jiangsu, China), 0.5 μL each of forward and reversed primers (10 μmol/L, 1 μL of cDNA, 8 μL of ddH_2_O). The reaction procedure was as follows: pre-denaturation at 95°C for 30 s; 95°C 10 s and 60°C 30 s, set 40 cycles; 95°C 15 s, 60°C 60 s, 95°C 15 s. For each RT-qPCR reaction, the assay was repeated 3 times for each sample. This was repeated 3 times for each test group. Relative quantification of total gene expression was calculated by 2^-ΔΔCt^. The GraphPad Prism 5.0 software was used to conduct multiple comparative analysis of the data. The data were expressed as mean ± standard deviation, and *p* < 0.05 was used as the criterion for differences that were significant.

### 2.6 Western blotting

Total protein was extracted from ovarian tissues of different reproductive cycles using RIPA buffer (Solarbio, R0010, Beijing, China), and protein concentrations were then determined using the BCA protein assay kit (Solarbio, PC0020, Beijing, China). Then SDS-PAGE loading buffer (Solarbio, P1016, Beijing, China) was added to the extracted protein sample (protein sample: loading buffer = 3: 1), the sample was boiled for 5 min, cooled to room temperature, centrifuged at 14,000 rpm for 5 min, and the supernatant was collected for subsequent experiments. Equal proteins were separated by 12% sodium dodecyl sulfate-polyacrylamide gel electrophoresis (SDS-PAGE). Then it was transferred to polyvinylidene fluoride (PVDF) film, immersed in 5% skim milk for 2 h at room temperature, and incubated with rabbit anti-CDO1 antibody (Bioss, bs-5808R, Beijing, China) and rabbit polyclonal antibody against beta actin (Affinity, AF7018, Jiangsu, China) at 4°C overnight. The membranes were washed with Tris buffered saline with Tween-20 (TBST) six times, for 5 min each time, and secondary antibody (Proteintech, SA00001-2, Wuhan, Hubei, China) conjugated with horseradish peroxidase (HRP) (1: 5000) incubated for 2 h at room temperature. The membranes were rewashed with TBST six times, the strips were exposed to ECL luminous solution (Affinity, KF8003, Jiangsu, China), and the strips were photographed using the Amersham Imager 600 system.

### 2.7 Immunohistochemistry

Yak ovarian tissues fixed in 4% paraformaldehyde for 24 h were removed, embedded in wax blocks, and then sectioned at 4 μm. The paraffin sections were deparaffinized with dimethyl benzene and dehydrated through graded concentrations of ethanol, then boiled in 0.01 mol/L sodium citrate buffer (pH 6.0) for 10 min for antigen retrieval, and then washed with phosphate-buffered saline (PBS) for three times for 5 min each time. The following experiments were performed according to the immunohistochemistry kit (Maxim, KIT-9720, Fuzhou, Fujian, China). After the PBS buffer was removed, endogenous peroxidase blocking agents were used for blocking, and treated at 37°C for 10 min; nonspecific staining inhibitor was added to reduce non-specific staining, and incubated at room temperature for 10 min; rabbit anti-CDO1 antibody (Bioss, bs-5808R, Beijing, China) was dropped on the sections, and the sections were laid flat in a moist box and incubated overnight at 4°C. Secondary antibodies was added and incubated for 10 min at room temperature. Finally, streptomyces antibiotic protein-peroxidase was added and incubated at room temperature for 10 min. The sections were stained with DAB substrate kit (Solarbio, DA1010, Beijing, China), the freshly prepared color developing solution was added, the staining time was controlled under the microscope, and then washed with sterile water. The sections were then counterstained with hematoxylin for 2–3 min, washed under running water until the sections turned blue, then dehydrated and sealed with neutral gum. Finally, they were photographed under a microscope to preserve the images.

### 2.8 Statistical analyses

Each experiment consisted of at least three independent replicates. Differences at *p* < 0.05 were considered statistically significant. Image analysis was performed using Image J software, and GraphPad Prism 8.0 software was utilized for graphing purposes. All data are expressed as mean ± SEM.

## 3 Results

### 3.1 Amplification and cloning of the yak CDO1 gene

**Figure 1:**
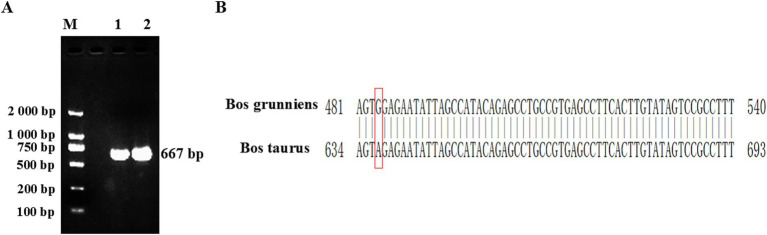
Cloning of *Bos grunniens* CDO1 gene. **(A)** Representative images of RT-PCR products separated by 1.5% agarose gel electrophoresis. M: DL2000 DNA Marker; 1–2: CDO1 amplification band in ovarian tissue (667 bp). **(B)** Difference of CDO1 gene sequence between *Bos grunniens* and *Bos taurus*. Base changes between *Bos grunniens* and *Bos taurus* are shown by the boxes.

PCR amplification was conducted using ovarian tissue cDNA as the template. The PCR amplification product was detected by 1.5% agar-gel electrophoresis. The length of the yak CDO1 gene amplification product was 667 bp, which was consistent with the expected size of the target fragment (Figure 1A). The results showed that the band of the amplified product was single, clear and bright, which met the requirements of the test and was suitable for further analysis. Through the splicing of DNAMAN software, we obtained the detailed base sequence of the CDO1 gene. The sequencing results were submitted to GenBank (GenBank: PP549430).

### 3.2 Bioinformatic analysis

#### 3.2.1 Sequence analysis of CDS region of the yak CDO1 gene

In this study, the sequence length of the cloned yak CDO1 gene was 667 bp, of which the CDS region was 603 bp. The base composition analysis showed that the base content was 28.3% A; 25.2% C; 25.2% G; 21.3% T. The cloned CDO1 gene sequence was compared with the CDS region of the CDO1 gene of *Bos taurus* (GenBank accession number: NM_001034465.2) published in NCBI. The similarity was 99.85%, and one base of the sequence was mutated (T > C) (Figure 1B). The online tool on NCBI was used to predict the cloned CDO1 gene sequence in the open reading frame. As shown in Table 3, the longest ORF was 603 bp, the start codon was located at 59 bp, and the stop codon was located at 661 bp, encoding a total of 200 amino acid residues.

**Table 3:** NCBI ORF finder result.

Label	Strand	Frame	Start	Stop	Length(nt | aa)
ORF1	+	2	59	661	603 | 200
ORF4	−	2	168	1	168 | 55
ORF3	−	1	604	473	132 | 43
ORF2	+	3	177	278	102 | 33
ORF5	−	3	374	273	102 | 33

#### 3.2.2 Homology analysis of the yak CDO1 gene and phylogenetic tree construction

**Figure 2:**
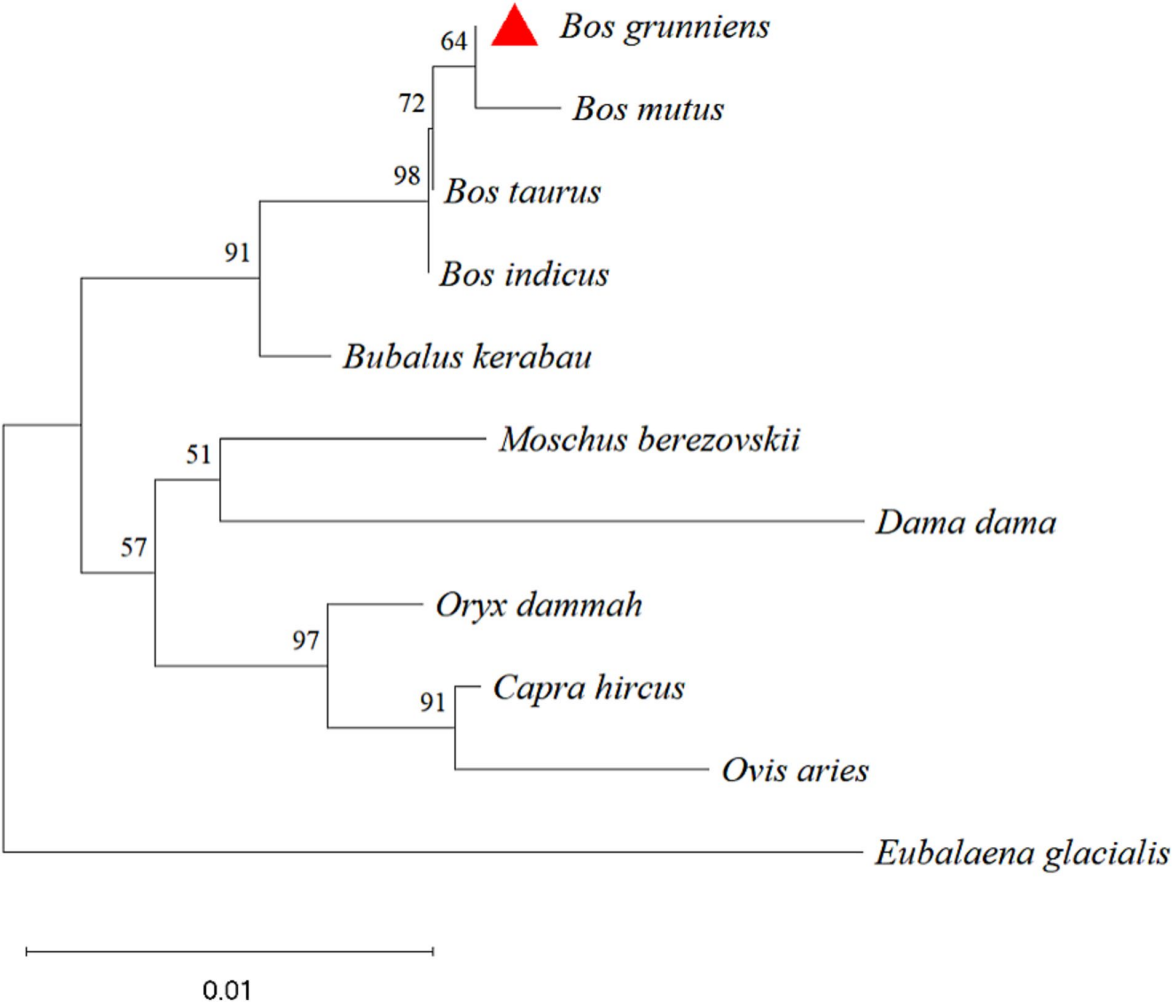
Phylogenetic tree based on nucleotide sequence of *Bos grunniens* CDO1 gene.

The nucleotide sequences of the yak CDO1 gene of yak were compared with those of *Bos taurus*, *Bos indicus*, *Bubalus kerabau*, *Bos mutus*, *Oryx dammah*, *Moschus berezovskii*, *Capra hircus*, *Ovis aries*, *Dama dama* and *Eubalaena glacialis* using DNAMAN software. The results showed that the homology was 99.85, 99.25, 98.95, 99.06, 97.45, 97.15, 97.15, 96.40, 95.63 and 93.84%, respectively. The similarity of the CDO1 gene was highest between *Bos grunniens* and *Bos taurus*. The lowest similarity was found in *Eubalaena glacialis* with only 93.84% (Table 4). According to the phylogenetic tree constructed by MEGA 11 software (Figure 2), *Bos grunniens* is clustered first with *Bos mutus*; then with *Bos taurus* and *Bos indicus*; and secondly with *Bubalus kerabau*. Among the 10 species compared, *Bos mutus* is the most closely related, and *Eubalaena glacialis* is the least losely related, indicating that it is relatively conservative in evolution.

**Table 4:** Nucleotide and amino acid homology comparisons of the yak CDO1 gene with that of other species.

Species	GenBank accession number	Nucleotide similarity /%	Amino acid Similarity /%
*Bos taurus*	NM_001034465.2	99.85	100.00
*Bos indicus*	XM_019968030.1	99.25	99.10
*Bubalus kerabau*	XM_055536543.1	98.95	99.55
*Bos mutus*	XM_005901940.2	99.06	99.53
*Oryx dammah*	XM_040225972.1	97.45	97.74
*Moschus berezovskii*	XM_055436649.1	97.15	97.74
*Capra hircus*	XM_005685043.3	97.15	90.95
*Ovis aries*	XM_015096777.3	96.40	90.95
*Dama dama*	XM_061158524.1	95.63	94.12
*Eubalaena glacialis*	XM_061188061.1	93.84	89.59

#### 3.2.3 Analysis of physicochemical properties and hydrophilicity of the encoded proteins

**Figure 3:**
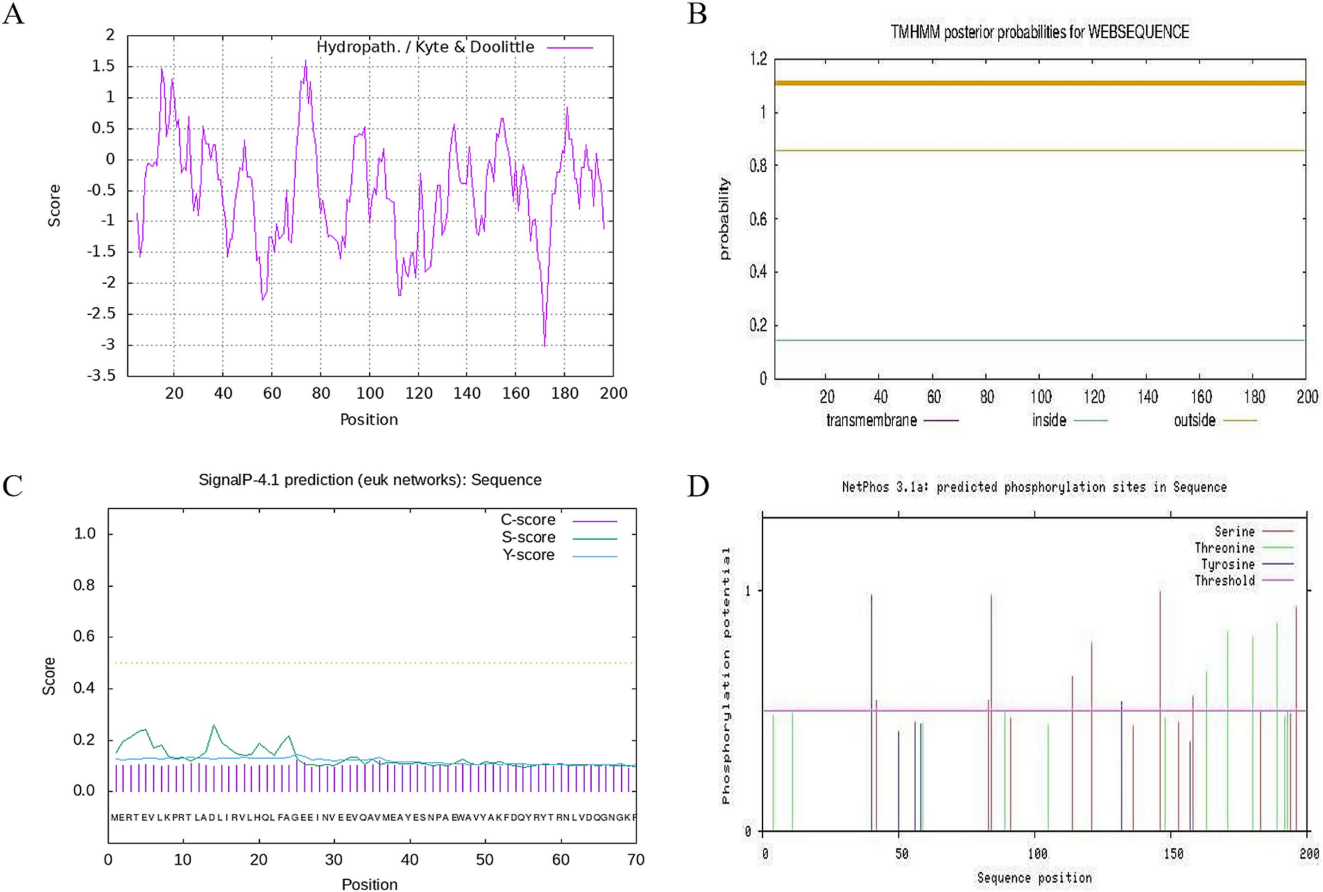
Hydrophobicity, transmembrane structure, signal peptide and phosphorylation site analysis of CDO1 protein. **(A)** Hydrophobicity analysis of CDO1 protein. **(B)** Prediction of transmembrane structure. **(C)** Signal peptide prediction. **(D)** Phosphorylation site analysis.

The results of the ProtParam analysis showed that CDO1 protein was composed of 200 amino acid residues and contained 20 amino acids. As shown in Table 5, glutamic acid and leucine were the most abundant, accounting for 8.5% of the total; tryptophan was the least abundant with 1.5%. The number of negatively charged amino acid residues (Asp+Glu) was 26, accounting for 13%, and 22 positively charged amino acid residues (Arg + Lys), accounting for 11%. The molecular formula of CDO1 is C_1020_H_1573_N_285_O_304_S_10_; the total number of atoms is 3,192; the molecular weight is 23.013 kDa; the theoretical isoelectric point (pI) is 6.25; the aliphatic index is 74.60; the estimated half-life is 30 h; the grand average of hydrophilicity (GRAVY) is −0.535; and it’s a hydrophilic protein. In addition, the hydrophilicity of the yak CDO1 protein was predicted by the Proscale online software (Figure 3A). The lower the hydrophilicity index, the more hydrophilic the protein. The isoleucine (73rd) and arginine (170th) were the most hydrophilic among the amino acid sequences encoding the proteins. The proportion of hydrophilic residues in the CDO1 protein is relatively high, which is consistent with the predictions of the online software ProtParam, indicating that the CDO1 protein is a hydrophilic protein.

**Table 5:** Amino acid composition of the CDO1 protein in *Bos grunniens*.

Amino acid	Quantity	Content /%	Amino acid	Quantity	Content /%
Alanine	11	5.5	Histidine	11	5.5
Phenylalanine	10	5.0	Leucine	17	8.5
Cysteine	4	2.0	Isoleucine	11	5.5
Aspartic acid	9	4.5	Lysine	13	6.5
Asparagine	13	6.5	Methionine	6	3.0
Glutamic acid	17	8.5	Proline	7	3.5
Glutamine	7	3.5	Arginine	9	4.5
Glycine	11	5.5	Serine	13	6.5
Threonine	12	6.0	Tryptophan	3	1.5
Valine	10	5.0	Tyrosine	6	3.0

#### 3.2.4 Subcellular localisation of encoded protein, transmembrane structure, signal peptide analysis

Prediction of subcellular localization using PSORT II online software showed that CDO1 protein was mainly located in the cytoplasm. TMHMM 2.0 analysis showed that CDO1 protein did not have a transmembrane helical structure and was located outside the cell, not a transmembrane protein (Figure 3B). SignalP4.1 analysis results showed that the S-score, C-score and Y-score are all below the threshold, so indicating that there is no signal peptide in this encoded protein (Figure 3C).

#### 3.2.5 Prediction of glycosylation site and potential phosphorylation site of CDO1 protein

NetOGlyc analysis showed that the O-glycosylation potential of threonine (Thr), the fourth amino acid in the CDO1 protein sequence, was greater than the threshold value, and was a potential O-glycosylation site. The phosphorylation site of CDO1 protein was analyzed using NetPhos 3.1 (Figure 3D). The results showed that 8 serine, 7 threonine and 2 tyrosine phosphorylation sites had potential values greater than the threshold value, and were potential phosphorylation sites in the sequence.

#### 3.2.6 Prediction of the secondary structure and tertiary structure of the encoded proteins

**Figure 4:**
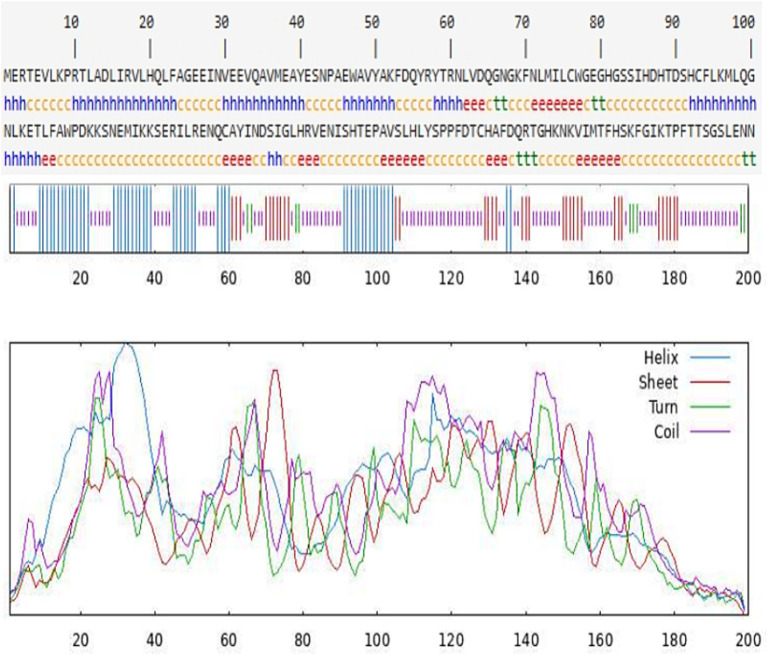
Prediction of CDO1 protein secondary structure.

**Figure 5:**
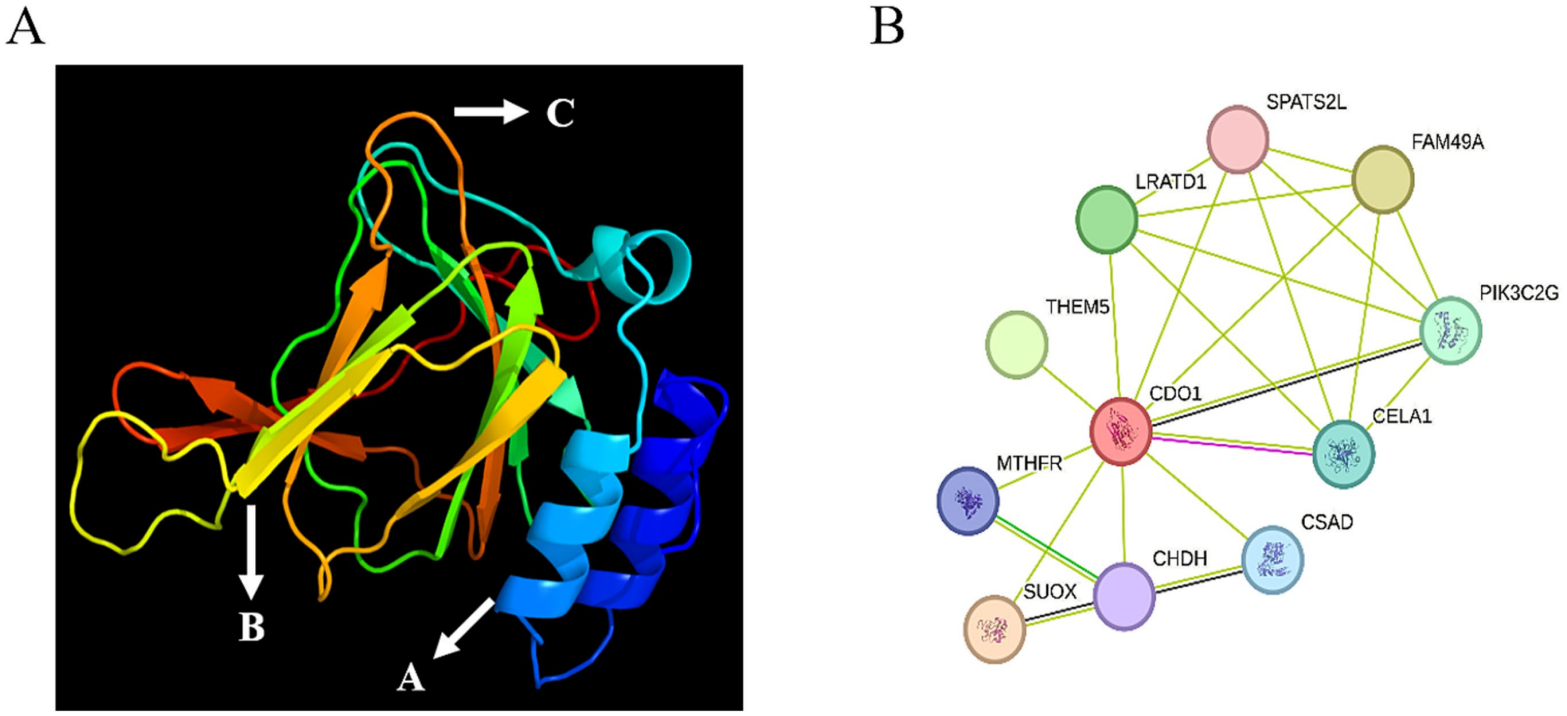
Tertiary structure and interaction network prediction of CDO1 protein. **(A)** Prediction of tertiary structure of CDO1 protein. A: Alpha helix; B: Extended strand; C: Random coil. **(B)** CDO1 protein interaction network.

The secondary structure of the CDO1 protein was predicted using the online software SOPMA (Figure 4). There are 55 *α*-helices, accounting for 27.50%; 34 extended strands, accounting for 17.00%; 9 *β*-turns, accounting for 4.50%; and 102 random coils, accounting for 51.00%. The tertiary structure of the CDO1 protein was predicted using the PHYRE2 online software (Figure 5A). The prediction results showed that the encoded protein is a double-stranded β-helix belonging to the RmlC-like cupins superfamily.

#### 3.2.7 Protein interaction network prediction

The online STRING software was used to predict the yak CDO1 interaction protein network. The results showed that the protein interaction network encoded by the CDO1 gene had 11 nodes and 23 edges (Figure 5B), the average degree of each node was 4.18, and the average local clustering coefficient was 0.89.

### 3.3 Relative expression of CDO1 in yak ovarian tissues during different reproductive cycles

**Figure 6:**
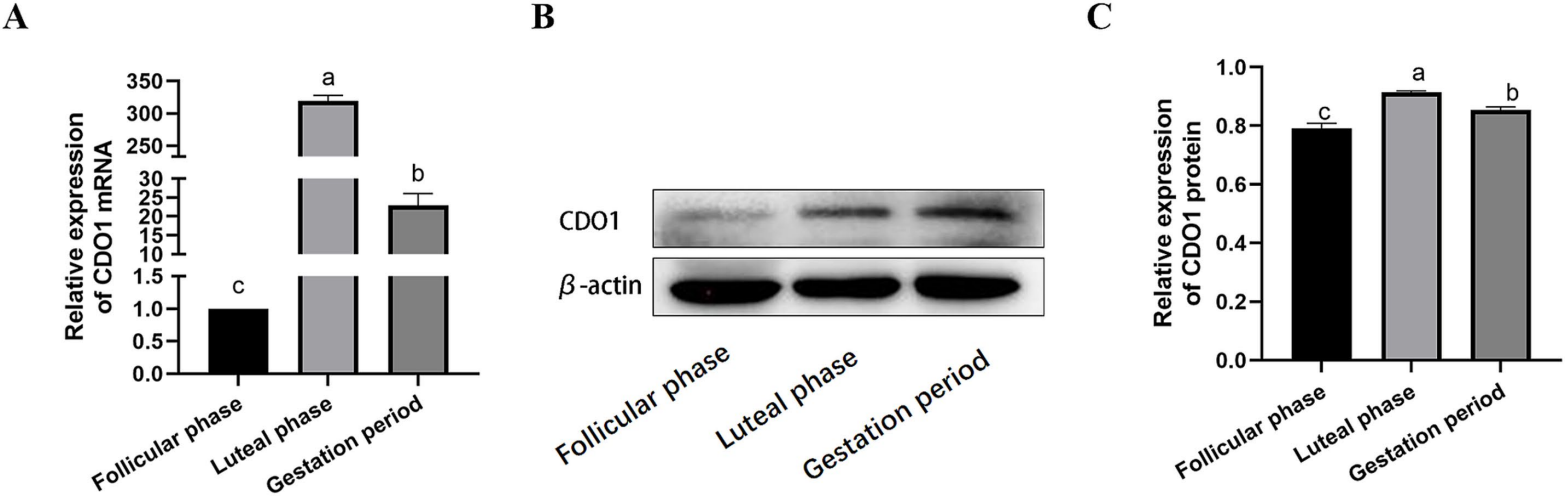
Expression of CDO1 mRNA and protein in ovarian tissues during different reproductive cycles. **(A)** The expression of CDO1 mRNA; **(B,C)** The expression of CDO1 protein. Significant differences (*p* < 0.05) are indicated by different letters.

The mRNA expression of the CDO1 gene was detected in the follicular phase, luteal phase and gestation period of the ovary in yaks using RT-qPCR (Figure 6A). The results demonstrated that ovarian expression was the most pronounced during the luteal phase, followed by the gestational period, and exhibited the lowest levels during the follicular phase (*p* < 0.01). Western blotting was used to detect CDO1 protein expression in yak ovaries at follicular, luteal and gestation stages (Figures 6B,C). The results were found to be in agreement with those obtained by RT-qPCR, with a statistically significant difference. (*p* < 0.01).

### 3.4 Localization of CDO1 in ovarian tissues of yaks during different reproductive cycles

**Figure 7:**
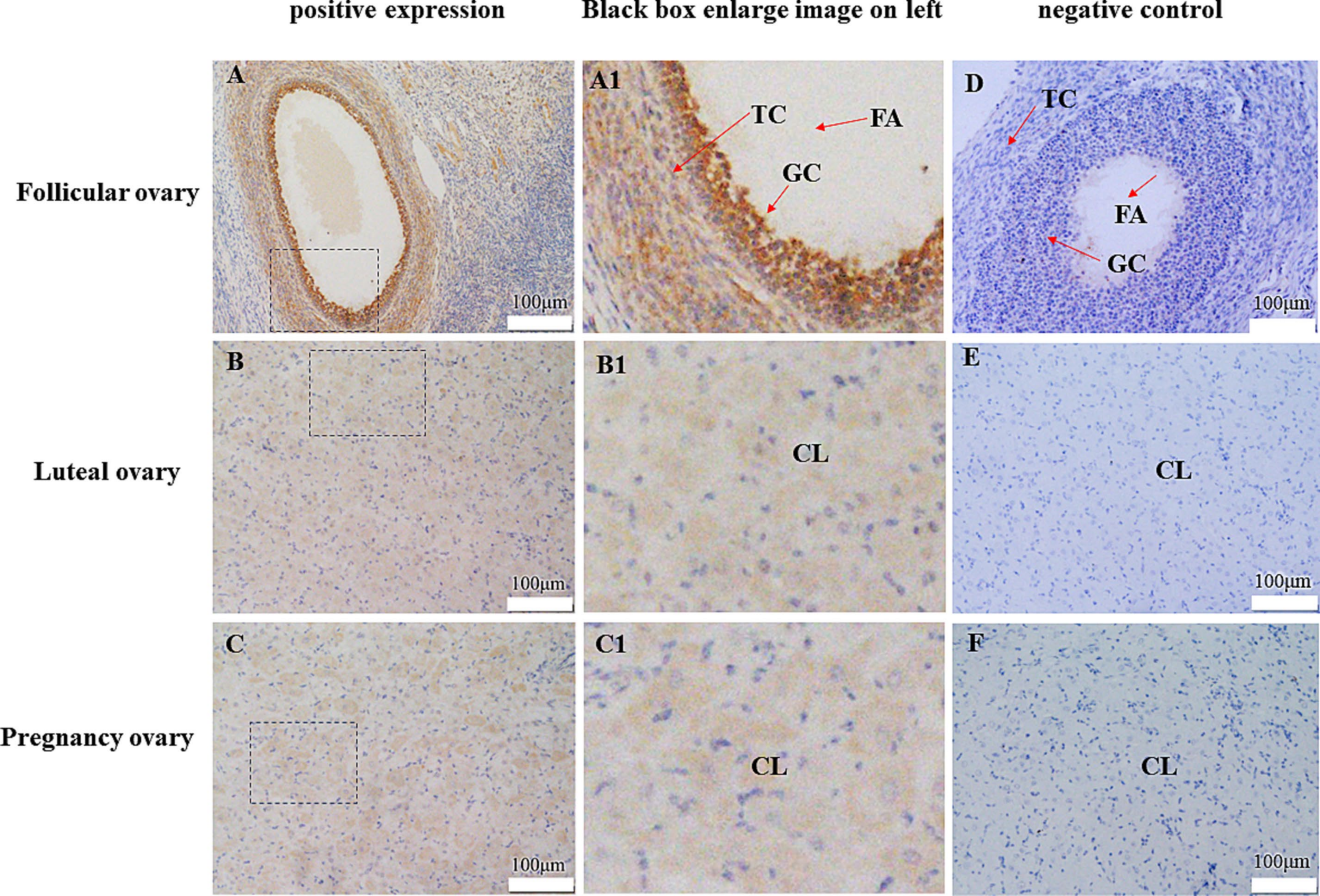
Representative immunohistochemical localization of CDO1 protein in follicular ovary, luteal ovary and pregnancy ovary. **(A)** Follicular ovary (positive expression). **(B)** Luteal ovary (positive expression). **(C)** Pregnancy ovary (positive expression). **(A1)** Enlarged image in the black box in A. **(B1)** Enlarged image in the black box in B. **(C1)** Enlarged image in the black box in C. **(D)** Follicular ovary (negative control). **(E)** Luteal ovary (negative control). **(F)** Pregnancy ovary (negative control). FA: antrum folliculi; GC: ovarian granular cells; TC: theca cell; CL: lutein cell.

Immunohistochemistry was used to detect the localization of CDO1 protein in yak ovarian tissue (Figure 7). The results showed that CDO1 protein was expressed in yak ovaries. In the follicular phase, CDO1 mainly located in granular cells and theca cells. During the luteal phase and pregnancy, it was mainly located in the lutein cells.

## 4 Discussion

Plateau environments are a major survival challenge for organisms at high altitudes such as the yak. As an ancient breed that inhabits the plateau, its reproductive capacity is constrained by the harsh environmental conditions, including intense ultraviolet radiation, low oxygen levels, and extreme temperatures. These factors collectively influence the number of fetuses it can produce (19). It has been shown that yaks have evolved unique physiological and genetic adaptive traits to overcome these environmental stresses (20). For example, at the molecular level, they exhibit up-regulated expression of genes associated with oxygen sensing and utilization (21–23), contributing to an increase in blood oxygen-carrying capacity; yak gut microbes influence the adaptive capacity of yaks, co-evolving with the host and helping the host to cope with challenges such as food shortage, phenotypic plasticity and adaptive immunity (24); in addition, many antioxidants can help yaks cope with oxidative stress and improve antioxidant performance (25). These adaptive changes not only safeguard the yak’s survival in the highlands, but also positively affect its reproductive success, especially playing a key role in maintaining pregnancy rates and foetal survival. One of the most notable physiological functions of taurine is anti-oxidative stress, and CDO1 is an important element for its synthesis. The study of the coding region of the yak CDO1 gene is important for understanding its resistance to adversity, thereby improving the yak’s ability to survive and reproduce in the face of adversity.

Therefore, in this study, the product of the yak CDO1 gene was cloned with a length of 667 bp and a CDS region of 603 bp, encoding 200 amino acids. The similarity of CDO1 gene sequences between *Bos grunniens* and 10 species showed that *Bos taurus* had the highest similarity of nucleotide and amino acid. Amino acid comparative analysis also showed that the CDO1 gene of the yak was more than 90% similar to that of other mammals, which was in line with the law of species evolution. Phylogenetic tree analysis shows that *Bos grunniens* is most closely related to *Bos mutus*, and the relationship with *Eubalaena glacialis* is the most distant, indicating that the CDO1 gene is highly conserved and species-specific in evolution. CDO1 has 17 potential phosphorylation sites, and protein post-translational modification includes phosphorylation, glycosylation, acetylation and other processing modes (26). It is speculated that these sites may be the key sites for post-translational modification of CDO1 protein to ensure the normal biological function of CDO1 protein in animals. It is a protein of the Cysteine dioxygenase type I family, which is presumed to be related to sulfur metabolism, especially the process of catalyzing cysteine oxidation. These prediction results will lay a foundation for further analysis of the function of the CDO1 protein and reveal its possible biological processes, as well as a theoretical basis for further exploration of the role of the CDO1 gene in yak reproduction and production.

In the predicted protein interaction network, each node represents the protein encoded by a single protein-coding gene, and the edge represents the interaction between proteins. According to the prediction of the online STRING software, the CDO1 protein can interact with 10 proteins. Cysteine sulfinic acid decarboxylase (CSAD) and CDO1 are involved in a series of enzymatic reactions in the synthesis of taurine. Taurine has many synthetic pathways, the most important being that cysteine is oxidized by CDO to cysteine sulfinic acid, and then decarboxylate becomes hypotaurine under the action of CSAD, and hypotaurine is oxidized to taurine. Cysteine can also be converted to sulfoalanine and then decarboxylated to taurine under the action of CSAD (27). In this synthetic pathway, CDO and CSAD are rate-limiting enzymes, also known as the CDO/CSAD pathway. Chymotrypsin-like elastase 1 (CELA1) is a serine protease whose function is to hydrolyze elastase (28). Down-regulation of CELA1 gene has been found in the primary tubal epithelial cells of porcine cultured *in vitro*, which is speculated to be involved in the growth and differentiation of tubal epithelial cells (29), providing new possibilities for advanced biotechnology of assisted reproduction. Methylenetetrahydrofolate reductase (MTHFR) plays a pivotal role in the metabolism of folate and methionine. Additionally, it is an indispensable enzyme for the metabolism of homocysteine, which is essential for the synthesis of taurine. It plays an important role in preventing cell dysfunction and maintaining intracellular environmental homeostasis (30, 31). By affecting the levels of folate and homocysteine in the body, MTHFR can cause a series of adverse pregnancy outcomes and gynecological diseases, such as recurrent abortion (32), gestational diabetes (33) and preeclampsia (34). In the process of normal blastocyst development, MTHFR in embryos is indispensable, and knockout of MTHFR gene can significantly reduce blastocyst rate, total blastocyst number, number of nourishing ectoderm and the quality of the inner cells (35). The analysis of the protein interaction network revealed that the aforementioned proteins are primarily involved in metabolic and synthetic processes, maintaining cellular homeostasis and influencing animal growth and reproduction.

The estrus of organisms varies periodically, and the main physiological activities in the follicular phase are oocyte discharge, endometrial proliferation and hypertrophy, and increased secretion activities of the deep glands (36). The period from the formation of the corpus luteum after ovulation to the onset of its degeneration is called the luteal phase (37, 38). During this period, progesterone secreted by the luteum acts on the endometrium to further develop, and the endometrium continues to thicken, providing physical and physiological conditions for embryo attachment (39). In gestation, yak ovaries continue to increase, and regulate individual estrus cycle and maintain pregnancy by secreting a large amount of progesterone, so as to ensure the normal reproductive ability of the body.

In order to further investigate the expression of CDO1 in yak ovaries, this study detected the expression of CDO1 in in the follicular, luteal and gestational phases. It was found that the expression trend of the gene and protein was consistent in different reproductive cycles, and the expression was highest in the luteal phase, followed by the gestation phase and finally the follicular phase. It is speculated that CDO1 plays an important role in enhancing endometrial receptivity, promoting hormone secretion and motivating embryo attachment. Studies have shown that CDO is involved in embryo attachment and development by affecting the secretion and synthesis of E_2_ and P_4_ (15). Immunohistochemical staining of ovarian tissues from yaks at different reproductive cycles showed that high levels of CDO1 were expressed in corpus luteum cells. The corpus luteum is a temporary endocrine gland rich in blood vessels, formed by the remaining follicles after ovulation. Its main function is to synthesize steroid hormones. The corpus luteum is mainly composed of two types of cells, the granulosa luteum cell (GLC) and the theca luteum cell (TLC), which are mainly responsible for secreting progesterone and synthesizing estrogen (40). CDO1 was also fully expressed in granular cells and theca cells at the follicular stage. Follicular granular cells and theca cells establish gap junction and desmosome junction through oocyte derivation, i.e., granular cells can provide nutrients and information factors to oocytes, thus promoting oocyte growth and maturation (41, 42). Therefore, CDO1 is involved in a number of physiological activities such as the regulation of the oestrus hormone in yaks and the exchange of information between cells to ensure the normal fertility and reproductive capacity of the body. However, its specific mechanism needs to be further investigated.

## 5 Conclusion

In this experiment, we successfully cloned the yak CDO1 gene with a product length of 667 bp and a CDS region of 603 bp, encoding a total of 200 amino acids. The CDO1 gene is relatively conserved in the evolutionary process, mainly distributed in the cytoplasm, and belongs to hydrophilic protein. CDO1 was expressed in ovarian tissues of yaks in different reproductive cycles, the highest in luteal stage, the second in the gestation stage and the lowest in the follicular stage. CDO1 may play an important role in regulating the maintenance of physiological function of yak ovarian tissue. Immunohistochemical results showed that CDO1 was mainly expressed in granular cells, theca cells and lutein cells of ovarian tissue of yaks, which provided basic data for further research on the mechanism of reproductive regulation in yak regulated by CDO1.

## Data Availability

The original contributions presented in the study are included in the article/Supplementary material, further inquiries can be directed to the corresponding author.

## Supplementary material

SUPPLEMENTARY FIGURE 1A Original images of RT-PCR products separated by 1.5% agarose gel electrophoresis. The leftmost band represents the DNA marker of 2000 bp. CDO1 amplification band in ovarian tissue is 667 bp.

SUPPLEMENTARY FIGURE 6A CDO1: The expression of CDO1 protein.

SUPPLEMENTARY FIGURE 6B *β*-actin: The expression of *β*-actin protein.

SUPPLEMENTARY FIGURE 7A Localization of CDO1 in yak follicular phase ovaries (positive expression).

SUPPLEMENTARY FIGURE 7b Localization of CDO1 in yak luteal phase ovaries (positive expression).

Supplementary Figure 7C Localization of CDO1 in yak ovaries during pregnancy (positive expression).

Supplementary Figure 7D Localization of CDO1 in yak follicular phase ovaries (negative control).

Supplementary Figure 7E Localization of CDO1 in yak luteal phase ovaries (negative control).

Supplementary Figure 7F Localization of CDO1 in yak ovaries during pregnancy (negative control).

## References

[1] Jiang H Chai ZX Chen XY Zhang CF Zhu Y Ji QM. Yak genome database: a multi-omics analysis platform. BMC Genomics. (2024) 25:346. doi: 10.1186/s12864-024-10274-6, PMID: 38580907 PMC10998334

[2] Ayalew W Chu M Liang C Wu X Yan P. Adaptation mechanisms of yak (*Bos grunniens*) to high-altitude environmental stress. Animals. (2021) 11:2344. doi: 10.3390/ani11082344, PMID: 34438801 PMC8388626

[3] Liu X Liu W Lenstra JA Zheng Z Wu X Yang J. Evolutionary origin of genomic structural variations in domestic yaks. Nat Commun. (2023) 14:5617. doi: 10.1038/s41467-023-41220-x, PMID: 37726270 PMC10509194

[4] Mo L Ma J Xiong Y Xiong X Lan D Li J. Factors influencing the maturation and developmental competence of yak (*Bos grunniens*) oocytes in vitro. Genes. (2023) 14:1882. doi: 10.3390/genes14101882, PMID: 37895231 PMC10606142

[5] Ma N He F Kawanokuchi J Wang G Yamashita T. Taurine and its anticancer functions: In Vivo and in vitro study. Adv Exp Med Biol. (2022) 1370:121–8. doi: 10.1007/978-3-030-93337-1_11 35882787

[6] Baliou S Adamaki M Ioannou P Pappa A Panayiotidis M Spandidos D. Protective role of taurine against oxidative stress (review). Mol Med Rep. (2021) 24:605. doi: 10.3892/mmr.2021.12242, PMID: 34184084 PMC8240184

[7] Naraki K Keshavarzi M Razavi BM Hosseinzadeh H. The protective effects of taurine, a non-essential amino acid, against metals toxicities: a review article. Biol Trace Elem Res. (2024):1–19. doi: 10.1007/s12011-024-04191-8 38735894

[8] Hirschberger LL Daval S Stover PJ Stipanuk MH. Murine cysteine dioxygenase gene: structural organization, tissue-specific expression and promoter identification. Gene. (2001) 277:153–61. doi: 10.1016/S0378-1119(01)00691-6, PMID: 11602353

[9] Paul BD Sbodio JI Snyder SH. Cysteine metabolism in neuronal Redox homeostasis. Trends Pharmacol Sci. (2018) 39:513–24. doi: 10.1016/j.tips.2018.02.007, PMID: 29530337 PMC5912966

[10] Tsuboyama-Kasaoka N Shozawa C Sano K Kamei Y Kasaoka S Hosokawa Y. Taurine (2-aminoethanesulfonic acid) deficiency creates a vicious circle promoting obesity. Endocrinology. (2006) 147:3276–84. doi: 10.1210/en.2005-1007, PMID: 16627576

[11] Guo YY Li BY Xiao G Liu Y Guo L Tang QQ. Cdo 1 promotes Pparγ-mediated adipose tissue lipolysis in male mice. Nat Metab. (2022) 4:1352–68. doi: 10.1038/s42255-022-00644-3, PMID: 36253617

[12] Chen M Zhu JY Mu WJ Guo L. Cysteine dioxygenase type 1 (Cdo 1): its functional role in physiological and pathophysiological processes. Genes Dis. (2023) 10:877–90. doi: 10.1016/j.gendis.2021.12.023, PMID: 37396540 PMC10308199

[13] Ueki I Roman HB Valli A Fieselmann K Lam J Peters R. Knockout of the murine cysteine dioxygenase gene results in severe impairment in ability to synthesize taurine and an increased catabolism of cysteine to hydrogen sulfide. Am J Physiol Endocrinol Metab. (2011) 301:E668–84. doi: 10.1152/ajpendo.00151.2011, PMID: 21693692 PMC3191547

[14] Asano A Roman HB Hirschberger LL Ushiyama A Nelson JL Hinchman MM. Cysteine dioxygenase is essential for mouse sperm osmoadaptation and male fertility. FEBS J. (2018) 285:1827–39. doi: 10.1111/febs.14449, PMID: 29604178 PMC5992081

[15] Zhang D Wang Z Luo X Guo H Qiu G Gong Y. Cysteine dioxygenase and taurine are essential for embryo implantation by involving in E (2)-Erα and P(4)-Pr signaling in mouse. J Anim Sci Biotechnol. (2023) 14:6. doi: 10.1186/s40104-022-00804-1, PMID: 36604722 PMC9814424

[16] Guerra DD Bok R Breen K Vyas V Jiang H MacLean KN. Estrogen regulates local cysteine metabolism in mouse myometrium. Reprod Sci. (2021) 28:79–90. doi: 10.1007/s43032-020-00284-6, PMID: 32820455

[17] Tao FY Ma HG Cao YQ Ji XY Song LM Xue P. Ameliorative effect of rare ginsenosides on reproductive injury induced by cyclophosphamide in female rats: based on metabonomics. Zhonghua Fu Chan Ke Za Zhi. (2024) 59:391–400. doi: 10.3760/cma.j.cn112141-20240116-00038, PMID: 38797569

[18] Behera A Sravanthi K Kumar LK Vedamurthy GV Singh D Onteru SK. Association of taurine with ovarian follicular steroids and postpartum anestrus condition in Murrah buffaloes. Domest Anim Endocrinol. (2021) 74:106511. doi: 10.1016/j.domaniend.2020.106511 32739763

[19] Ahmad HI Mahmood S Hassan M Sajid M Ahmed I Shokrollahi B. Author correction: genomic insights into yak (*Bos grunniens*) adaptations for nutrient assimilation in high-altitudes. Sci Rep. (2024) 14:18081. doi: 10.1038/s41598-024-68548-8, PMID: 39103510 PMC11300459

[20] Ahmad HI Mahmood S Hassan M Sajid M Ahmed I Shokrollahi B. Genomic insights into yak (*Bos grunniens*) adaptations for nutrient assimilation in high-altitudes. Sci Rep. (2024) 14:5650. doi: 10.1038/s41598-024-55712-3, PMID: 38453987 PMC10920680

[21] Ma X Wang M Wang J Han X Yang X Zhang H. Hypoxia-inducible factor 1α affects yak oocyte maturation and early embryonic development by regulating autophagy. Antioxidants. (2024) 13:840. doi: 10.3390/antiox13070840, PMID: 39061908 PMC11273763

[22] Ding X Yang C Bao P Wu X Pei J Yan P. Population genetic variations of the Mmp3 gene revealed hypoxia adaptation in domesticated yaks (*Bos grunniens*). Asian Australas J Anim Sci. (2019) 32:1801–8. doi: 10.5713/ajas.17.0706, PMID: 30381735 PMC6819682

[23] Bao Q Zhang X Bao P Liang C Guo X Chu M. Using weighted gene co-expression network analysis (Wgcna) to identify the hub genes related to hypoxic adaptation in yak (*Bos grunniens*). Genes Genomics. (2021) 43:1231–46. doi: 10.1007/s13258-021-01137-5, PMID: 34338989

[24] Wang R Bai B Huang Y Degen A Mi J Xue Y. Yaks are dependent on gut microbiota for survival in the environment of the Qinghai Tibet plateau. Microorganisms. (2024) 12:1122. doi: 10.3390/microorganisms12061122, PMID: 38930503 PMC11205922

[25] Jiang X Ma Y Gong S Zi X Zhang D. Resveratrol promotes proliferation, antioxidant properties, and progesterone production in yak (*Bos grunniens*) granulosa cells. Animals. (2024) 14:240. doi: 10.3390/ani14020240, PMID: 38254409 PMC10812796

[26] Qualmann B Kessels MM. The role of protein arginine methylation as post-translational modification on actin cytoskeletal components in neuronal structure and function. Cells. (2021) 10:1079. doi: 10.3390/cells10051079, PMID: 34062765 PMC8147392

[27] Wen C Li F Zhang L Duan Y Guo Q Wang W. Taurine is involved in energy metabolism in muscles, adipose tissue, and the liver. Mol Nutr Food Res. (2019) 63:e1800536. doi: 10.1002/mnfr.201800536, PMID: 30251429

[28] Ojha M Smith NJ Devine AJ Joshi R Goodman EM Fan Q. Anti-Cela1 antibody Kf4 prevents emphysema by inhibiting stretch-mediated remodeling. JCI Insight. (2024) 9:e169189. doi: 10.1172/jci.insight.169189, PMID: 38193533 PMC10906462

[29] Kulus M Kranc W Wojtanowicz-Markiewicz K Celichowski P Światły-Błaszkiewicz A Matuszewska E. New gene markers expressed in porcine Oviductal epithelial cells cultured primary in vitro are involved in ontological groups representing physiological processes of porcine oocytes. Int J Mol Sci. (2021) 22:2082. doi: 10.3390/ijms22042082, PMID: 33669854 PMC7923230

[30] Blomgren LKM Huber M Mackinnon SR Bürer C Baslé A Yue WW. Dynamic inter-domain transformations mediate the allosteric regulation of human 5, 10-methylenetetrahydrofolate reductase. Nat Commun. (2024) 15:3248. doi: 10.1038/s41467-024-47174-y, PMID: 38622112 PMC11018872

[31] Raghubeer S Matsha TE. Methylenetetrahydrofolate (Mthfr), the one-carbon cycle, and cardiovascular risks. Nutrients. (2021) 13:4562. doi: 10.3390/nu13124562, PMID: 34960114 PMC8703276

[32] Alfaleh A Alkattan A Mahmoud N Alfaleh F Almutair N Alanazi A. The association between Mthfr C677T gene polymorphism and repeated pregnancy loss in Arabic countries: a systematic review and Meta-analysis. Reprod Sci. (2023) 30:2060–8. doi: 10.1007/s43032-023-01201-3, PMID: 36854824

[33] Williamson JM Arthurs AL Smith MD Roberts CT Jankovic-Karasoulos T. High folate, perturbed one-carbon metabolism and gestational diabetes mellitus. Nutrients. (2022) 14:3930. doi: 10.3390/nu14193930, PMID: 36235580 PMC9573299

[34] Umapathy A Chamley LW James JL. Reconciling the distinct roles of angiogenic/anti-angiogenic factors in the placenta and maternal circulation of normal and pathological pregnancies. Angiogenesis. (2020) 23:105–17. doi: 10.1007/s10456-019-09694-w, PMID: 31707538

[35] Ishitani H Ikeda S Egashira K Sugimoto M Kume S Minami N. Embryonic Mthfr contributes to blastocyst development. J Assist Reprod Genet. (2020) 37:1807–14. doi: 10.1007/s10815-020-01898-0, PMID: 32767205 PMC7468012

[36] Alfattah MA Correia CN Browne JA McGettigan PA Pluta K Carrington SD. Transcriptomics analysis of the bovine endometrium during the perioestrus period. PLoS One. (2024) 19:e0301005. doi: 10.1371/journal.pone.0301005 38547106 PMC10977793

[37] Yang X Gao S Luo W Fu W Xiong Y Li J. Dynamic transcriptome analysis of Maiwa yak corpus luteum during the estrous cycle. Anim Biotechnol. (2023) 34:4569–79. doi: 10.1080/10495398.2023.2174130, PMID: 36752221

[38] Grazul-Bilska AT Dorsam ST Reyaz A Valkov V Bass CS Kaminski SL. Follicle-stimulating hormone receptors expression in ovine corpora lutea during luteal phase: effect of nutritional plane and follicle-stimulating hormone treatment. Domest Anim Endocrinol. (2020) 71:106391. doi: 10.1016/j.domaniend.2019.106391, PMID: 31731250

[39] Złotkowska A Andronowska A. Modulatory effect of chemokines on porcine endometrial stromal and endothelial cells. Domest Anim Endocrinol. (2020) 72:106475. doi: 10.1016/j.domaniend.2020.106475 32371294

[40] Przygrodzka E Plewes MR Davis JS. Luteinizing hormone regulation of inter-organelle communication and fate of the Corpus luteum. Int J Mol Sci. (2021) 22:9972. doi: 10.3390/ijms22189972, PMID: 34576135 PMC8470545

[41] Richani D Dunning KR Thompson JG Gilchrist RB. Metabolic co-dependence of the oocyte and cumulus cells: essential role in determining oocyte developmental competence. Hum Reprod Update. (2021) 27:27–47. doi: 10.1093/humupd/dmaa043, PMID: 33020823

[42] Xie J Xu X Liu S. Intercellular communication in the cumulus-oocyte complex during folliculogenesis: a review. Front Cell Dev Biol. (2023) 11:1087612. doi: 10.3389/fcell.2023.1087612, PMID: 36743407 PMC9893509

