# Supplementary figures and images for: Cloning, bioinformatics analysis and expression of the cysteine dioxygenase type 1 (CDO1) gene in domestic yak
Source: Front Vet Sci. 2024 Oct 18;11:1488782. doi: 10.3389/fvets.2024.1488782 (PMC11527789; doi:10.3389/fvets.2024.1488782)

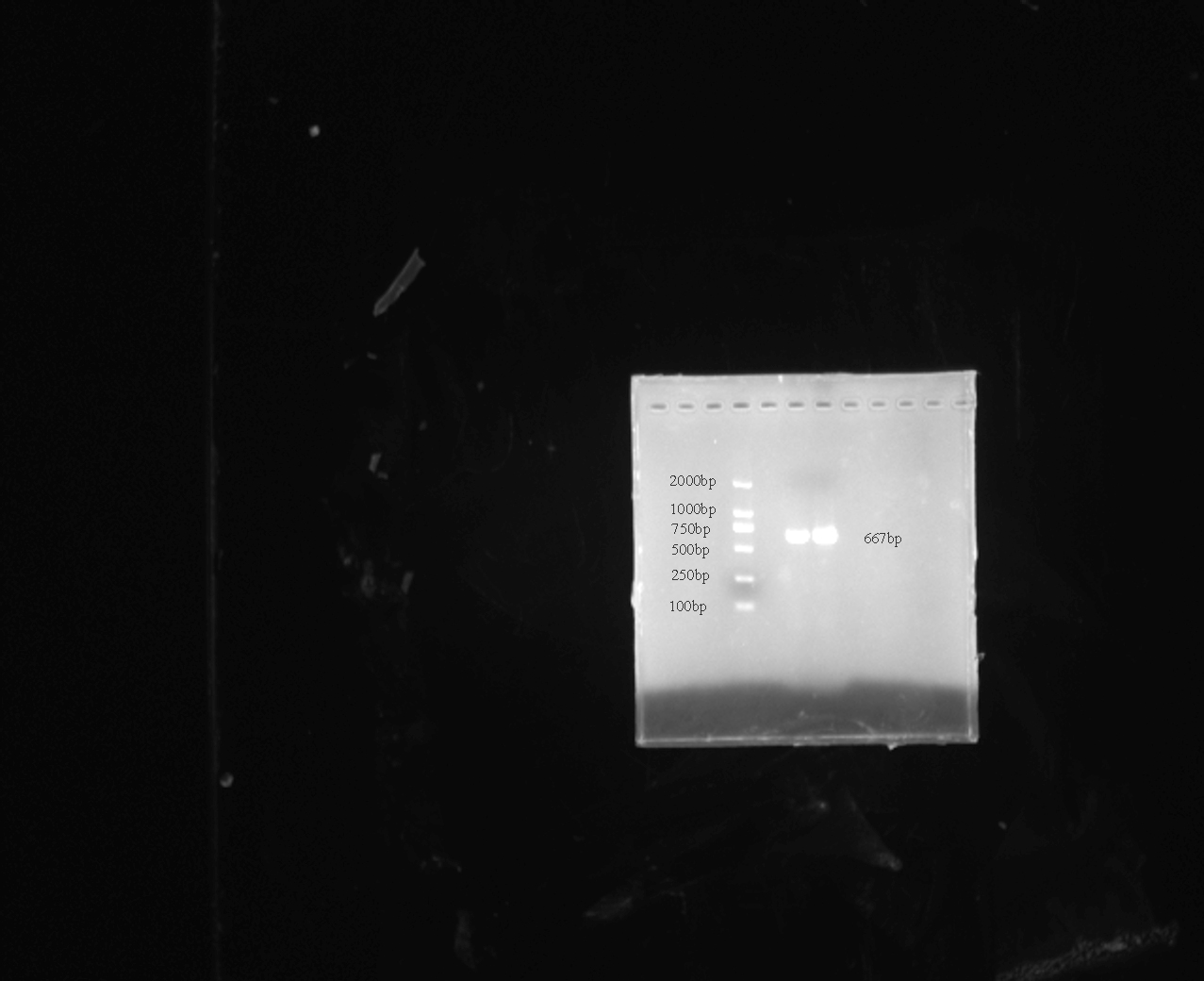

Supplement: SUPPLEMENTARY FIGURE 1A — Original images of RT-PCR products separated by 1.5% agarose gel electrophoresis. The leftmost band represents the DNA marker of 2000 bp. CDO1 amplification band in ovarian tissue is 667 bp. [file Image_1.JPEG]

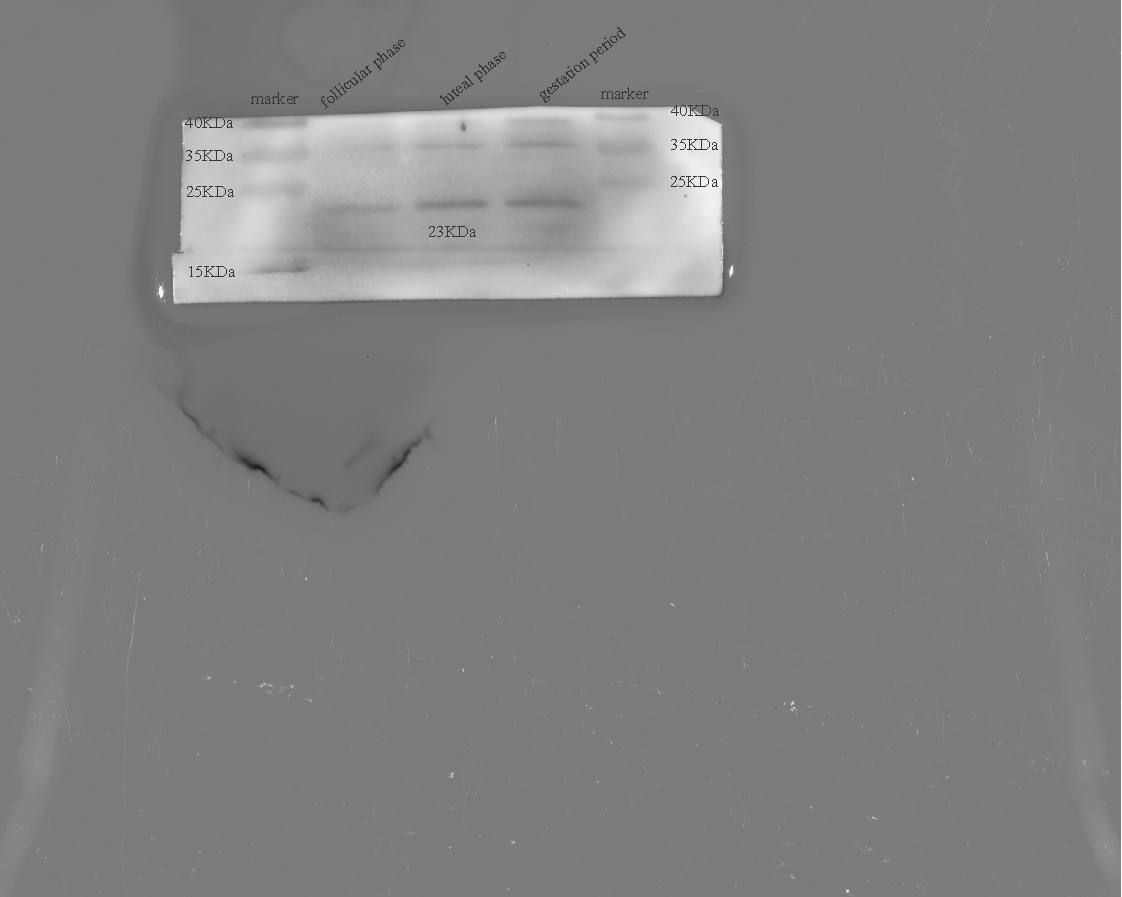

Supplement: SUPPLEMENTARY FIGURE 6A — CDO1: The expression of CDO1 protein. [file Image_2.JPEG]

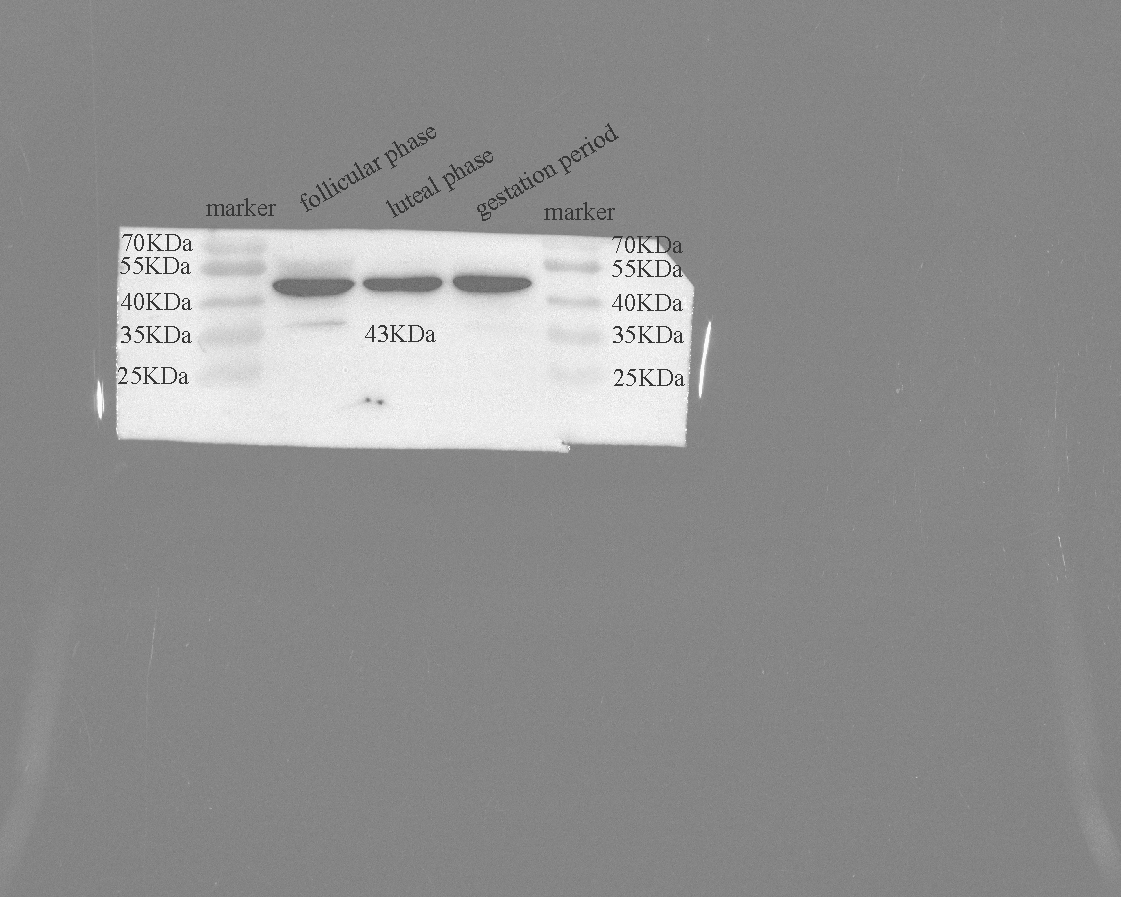

Supplement: SUPPLEMENTARY FIGURE 6B — β-actin: The expression of β-actin protein. [file Image_3.JPEG]

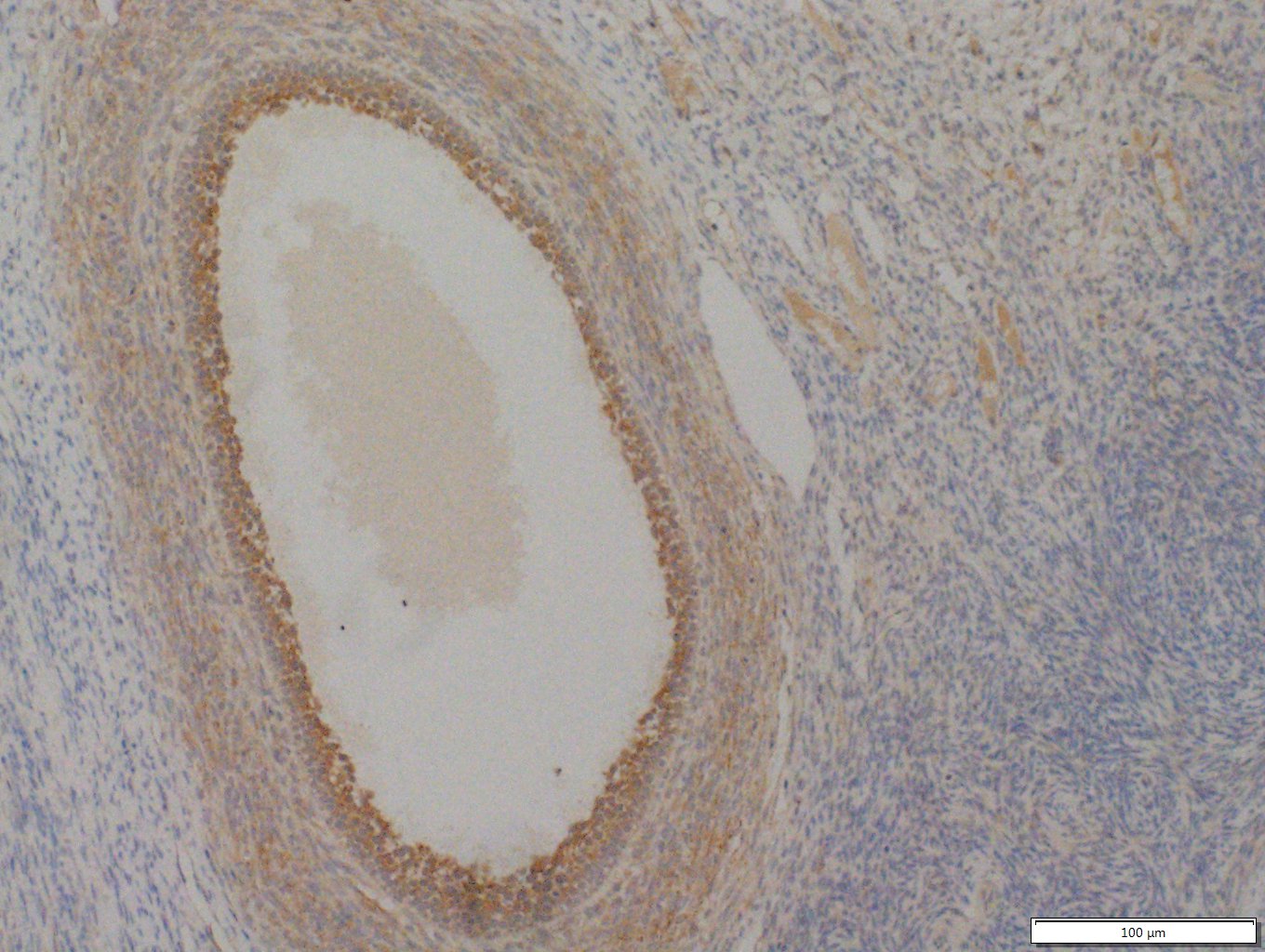

Supplement: SUPPLEMENTARY FIGURE 7A — Localization of CDO1 in yak follicular phase ovaries (positive expression). [file Image_4.JPEG]

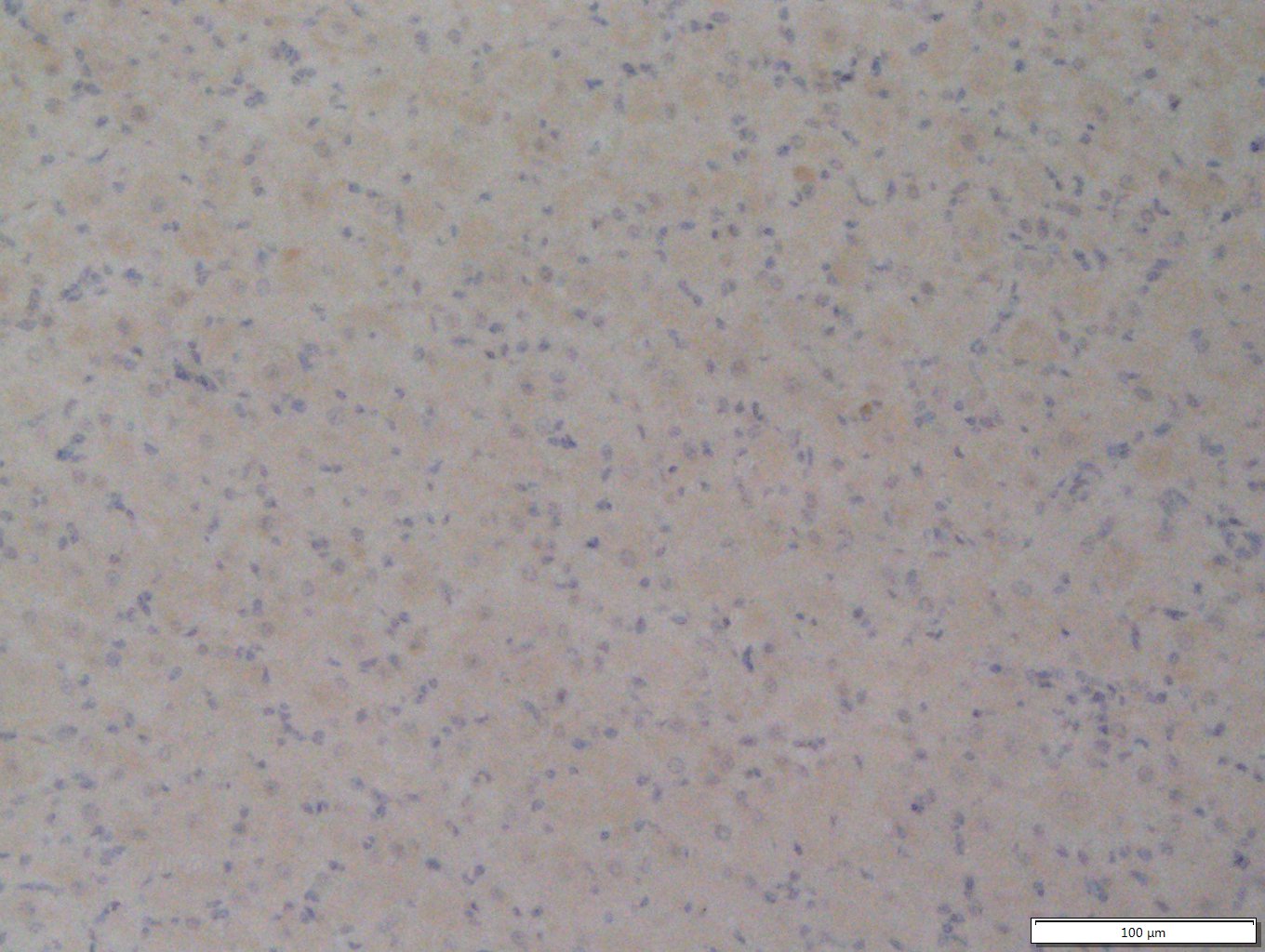

Supplement: SUPPLEMENTARY FIGURE 7b — Localization of CDO1 in yak luteal phase ovaries (positive expression). [file Image_5.JPEG]

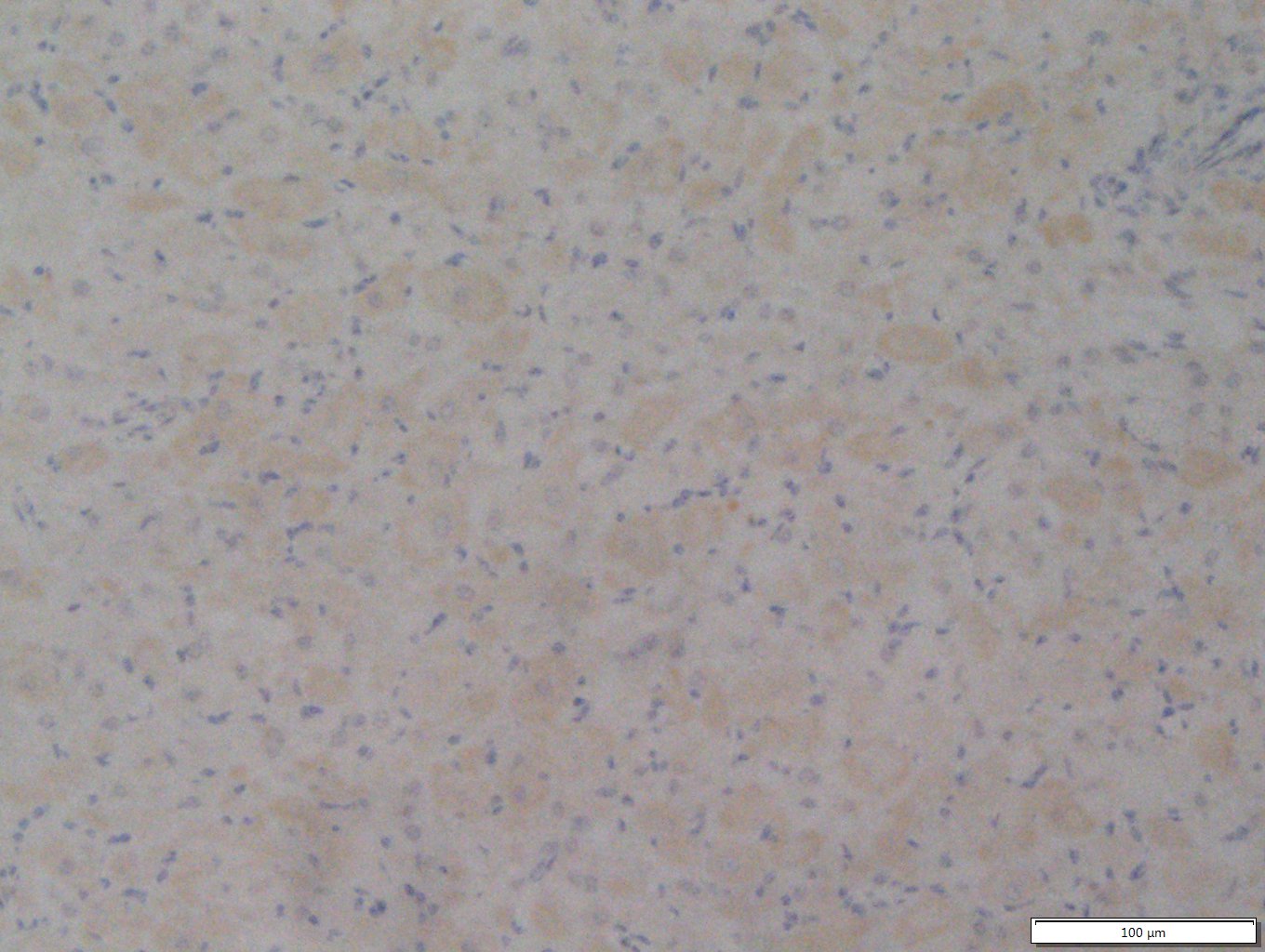

Supplement: Supplementary Figure 7C — Localization of CDO1 in yak ovaries during pregnancy (positive expression). [file Image_6.JPEG]

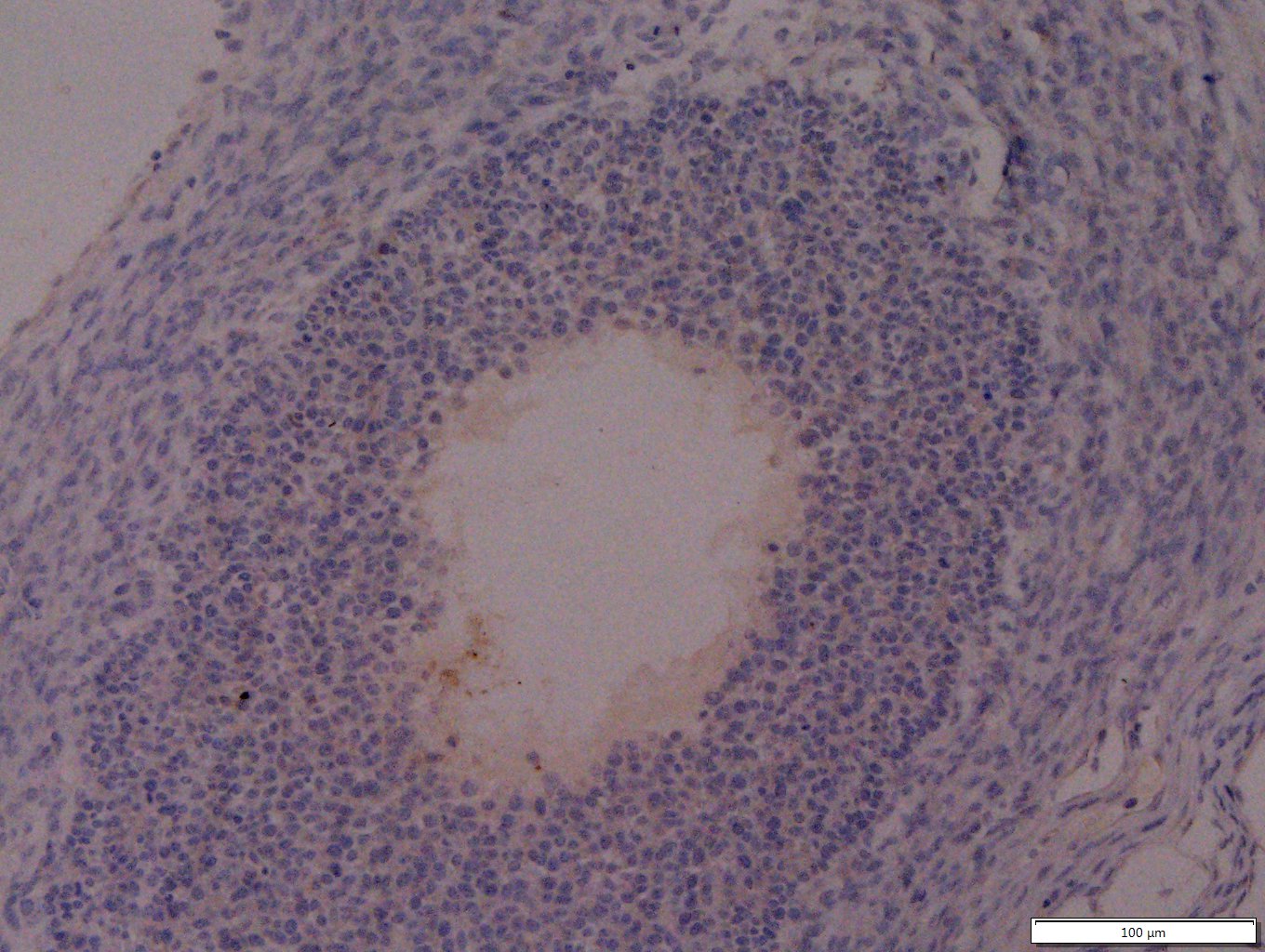

Supplement: Supplementary Figure 7D — Localization of CDO1 in yak follicular phase ovaries (negative control). [file Image_7.JPEG]

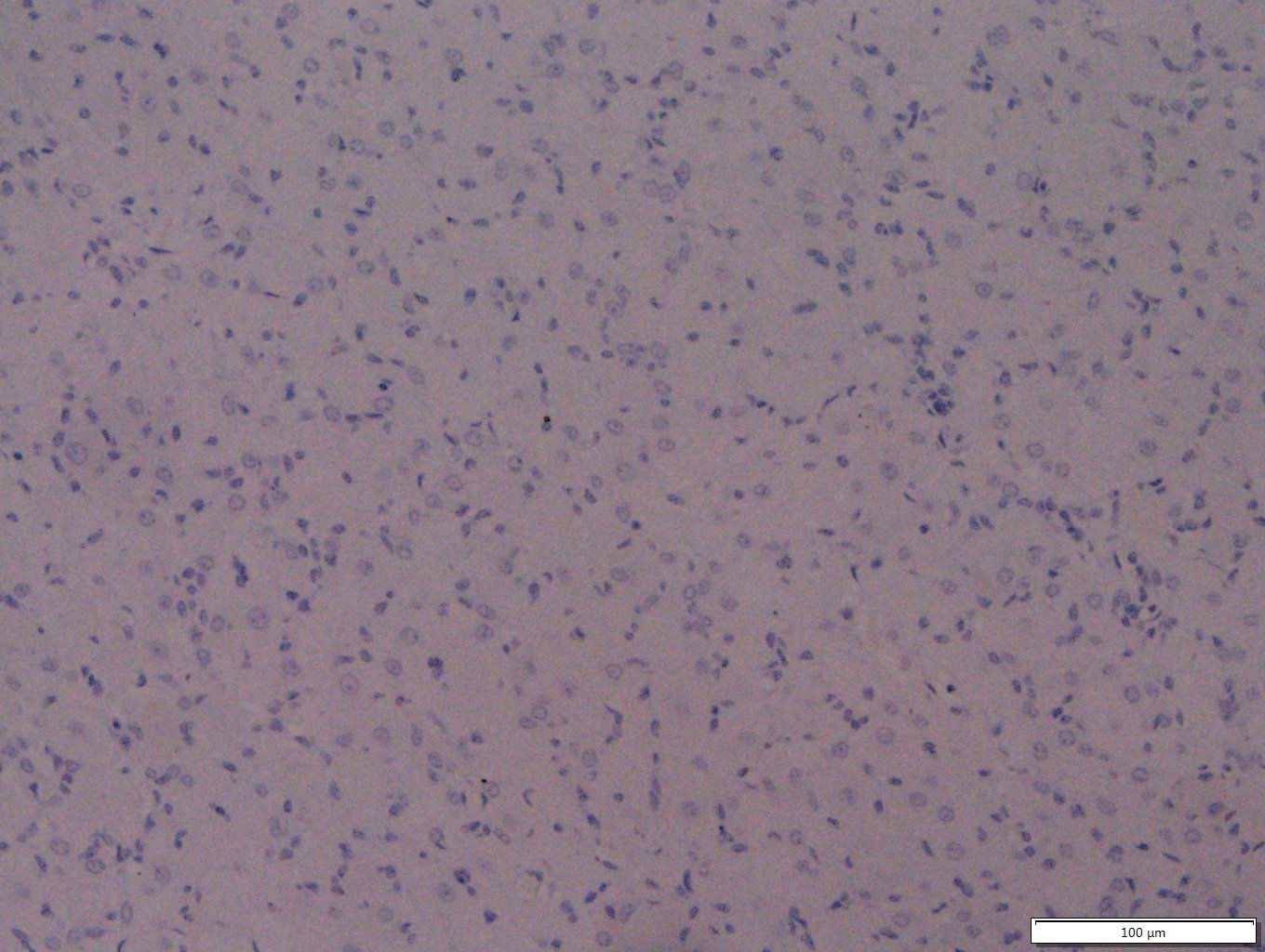

Supplement: Supplementary Figure 7E — Localization of CDO1 in yak luteal phase ovaries (negative control). [file Image_8.JPEG]

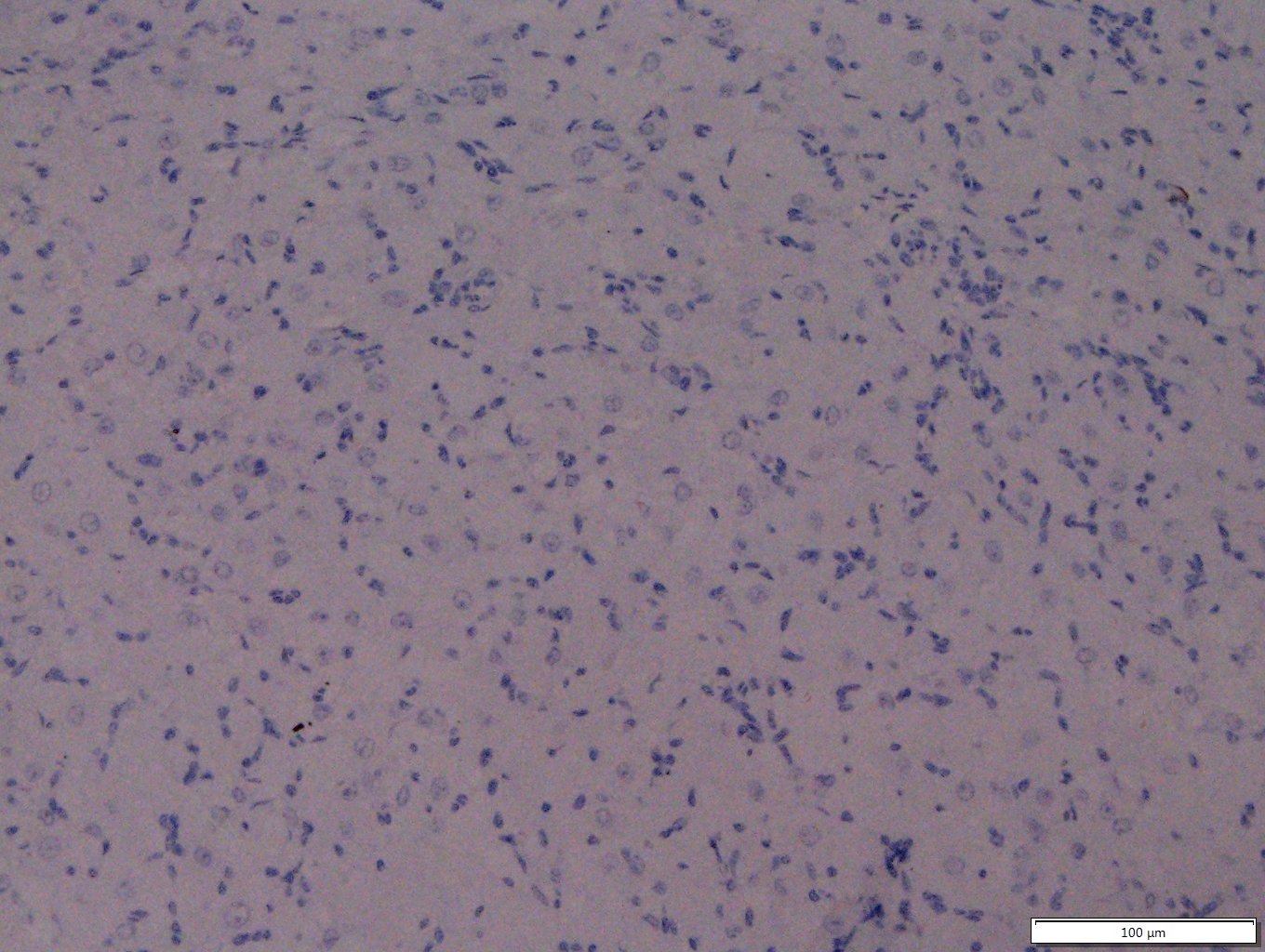

Supplement: Supplementary Figure 7F — Localization of CDO1 in yak ovaries during pregnancy (negative control). [file Image_9.JPEG]
